# Influence of Volatile Anesthesia on the Release of Glutamate and other Amino Acids in the Nucleus Accumbens in a Rat Model of Alcohol Withdrawal: A Pilot Study

**DOI:** 10.1371/journal.pone.0169017

**Published:** 2017-01-03

**Authors:** Thomas Seidemann, Claudia Spies, Rudolf Morgenstern, Klaus-Dieter Wernecke, Nicolai Netzhammer

**Affiliations:** 1 Department of Anesthesiology and Intensive Care Medicine, Campus Charité Mitte and Campus Virchow-Klinikum, Charité – Universitätsmedizin Berlin, Berlin, Germany; 2 Institute of Pharmacology, Campus Charité Mitte, Charité – Universitätsmedizin Berlin, Berlin, Germany; 3 SOSTANA, Berlin, Germany; Albany Medical College, UNITED STATES

## Abstract

**Background:**

Alcohol withdrawal syndrome is a potentially life-threatening condition, which can occur when patients with alcohol use disorders undergo general anesthesia. Excitatory amino acids, such as glutamate, act as neurotransmitters and are known to play a key role in alcohol withdrawal syndrome. To understand this process better, we investigated the influence of isoflurane, sevoflurane, and desflurane anesthesia on the profile of excitatory and inhibitory amino acids in the nucleus accumbens (NAcc) of alcohol-withdrawn rats (AWR).

**Methods:**

Eighty Wistar rats were randomized into two groups of 40, pair-fed with alcoholic or non-alcoholic nutrition. Nutrition was withdrawn and microdialysis was performed to measure the activity of amino acids in the NAcc. The onset time of the withdrawal syndrome was first determined in an experiment with 20 rats. Sixty rats then received isoflurane, sevoflurane, or desflurane anesthesia for three hours during the withdrawal period, followed by one hour of elimination. Amino acid concentrations were measured using chromatography and results were compared to baseline levels measured prior to induction of anesthesia.

**Results:**

Glutamate release increased in the alcohol group at five hours after the last alcohol intake (p = 0.002). After 140 min, desflurane anesthesia led to a lower release of glutamate (p < 0.001) and aspartate (p = 0.0007) in AWR compared to controls. GABA release under and after desflurane anesthesia was also significantly lower in AWR than controls (p = 0.023). Over the course of isoflurane anesthesia, arginine release decreased in AWR compared to controls (p < 0.001), and aspartate release increased after induction relative to controls (p_20min_ = 0.015 and p_40min_ = 0.006). However, amino acid levels did not differ between the groups as a result of sevoflurane anesthesia.

**Conclusions:**

Each of three volatile anesthetics we studied showed different effects on excitatory and inhibitory amino acid concentrations. Under desflurane anesthesia, both glutamate and aspartate showed a tendency to be lower in AWR than controls over the whole timecourse. The inhibitory amino acid arginine increased in AWR compared to controls, whereas GABA levels decreased. However, there were no significant differences in amino acid concentrations under or after sevoflurane anesthesia. Under isoflurane, aspartate release increased in AWR following induction, and from 40 min to 140 min arginine release in controls was elevated. The precise mechanisms through which each of the volatile anesthetics affected amino acid concentrations are still unclear and further experimental research is required to draw reliable conclusions.

## Introduction

Alcohol use disorder is one of the most common causes of critical illness [1], with a lifetime prevalence of alcohol abuse in US adults of around 18% [2]. A quarter of patients with long term alcohol abuse will develop alcohol withdrawal syndrome (AWS) [3]. AWS is responsible for many comorbidities, including chronic disorders [4], and can lead to delirium tremens, a potentially life-threatening condition [5]. Alcohol interacts with a variety of protein targets in the central nervous system, including the glutamatergic, noradrenergic, dopaminergic, serotonergic, GABAergic and cholingergic receptor systems [6,7].

The nucleus accumbens (NAcc) is critical to the neocortical modulation of neurotransmitters. It plays a central role of the cortico-striato-thalamic-cortical-loop, using both excitatory and inhibitory transmitters to regulate signal transduction. Under physiological conditions, these excitatory and inhibitory systems are in balance. However, chronic alcohol consumption lowers glutamate levels in several regions of the brain including the striatum [7], which is important in mediating the alcohol withdrawal response [8]. Chronic alcohol intake also increases N-Methyl-D-aspartate (NMDA) receptor expression [9], and increased glutamate release following alcohol withdrawal results in an excessive excitatory activation via upregulated NMDA receptors. These changes are postulated to be responsible for causing the typical AWS symptoms, such as seizures and hyperexcitability [10].

When patients with alcohol use disorder have to undergo general anesthesia, alcohol withdrawal is often inevitable. Reports suggest that every fourth patient admitted to a surgical ward is positive for alcoholism [11], and that over the course of a stay at a postoperative intensive care unit, up to 35% of all patients show AWS [12].

General anesthetic agents elevate the activity of inhibitory neurotransmitter systems and decrease excitatory transmitter levels. However, it is unknown at present whether volatile anesthetics attenuate the increased excitatory amino acid levels seen in AWS.

Therefore, the aim of this pilot study was to investigate the influence of isoflurane, sevoflurane, and desflurane anesthesia on important excitatory and inhibitory amino acids in the NAcc of alcohol-withdrawn rats (AWR), which are an animal model of AWS.

## Materials and Methods

### Animals and treatments

**Fig 1:**
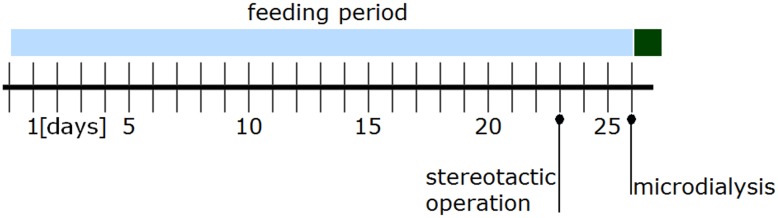
Schedule for the feeding period.

Eighty male rats (Wistar Unilever Outbred Rat; Stocks: HsdCpb:WU; Harlan Winkelmann, Borchen, Germany) were used according to experimental protocol G 0299/08. All procedures were approved by the institutional animal care board of the Landesamt für Gesundheit und Soziales (LAGeSo) in Berlin, Germany. The rats were housed in single cages under standard conditions (22 ± 1°C, day phase from 6 am to 6 pm), and habituated to manual handling for one week before the alcohol uptake period started. A two-bottle choice method, which is well established and frequently used by other study groups [13], was used to induce voluntary alcohol dependence in the rats. We modified the method to permit continuous rather than intermittent access, as other studies have done [14]. This allowed the first withdrawal response to occur during the experimental trial. The animals were randomized into two groups of 40, which were fed with either an alcoholic liquid nutrition (alcohol or treatment group) or a non-alcoholic liquid nutrition (control group) [15]. Apart from their different liquid diets, both groups received the same treatment throughout the experiment. Ethanol was added to the liquid diet to obtain a 5% concentration (v/v). In the non-alcoholic nutrition, isocaloric maltodextrin was added in place of ethanol, at the same concentration. Water was provided ad libitum, and liquid nutrition was pair-fed to the rats for 26 days (Fig 1) to give long enough for a sufficient withdrawal response to develop [16]. Prior studies with oral alcohol feeding protocols have shown that concentrations of 100–200 mg/dl are sufficient to induce alcohol addiction in rats [13,17,18]. After the 26 days, liquid food was withheld from both groups, in order to induce an alcohol withdrawal response in the alcohol group. Microdialysis trials were performed to measure the levels of amino acid neurotransmitters in the NAcc during the withdrawal response.

### In vivo microdialysis in freely moving rats

Microdialysis trials were performed as previously described [19]. On the 23^rd^ day of the feeding protocol (Fig 1), microdialysis cannulae were implanted in the NAcc according to the atlas of Paxinos and Watson using a stereotactic apparatus [20]. Each animal was anesthetized via intramuscular anesthesia using 120 mg/kg ketamine (Ketavet^®^, 100 mg/mL, Pfizer, New York, New York) and 5 mg/kg xylazine (Rompun^®^ 2%, Bayer, Leverkusen, Germany). The anesthetized rat was placed on the stereotactic apparatus to allow implantation of a microdialysis cannula above the right NAcc core region according to the following coordinates: anterior 0.12 cm, lateral 0.14 cm, and ventral -0.55 cm, relative to bregma and the dura. The cannula was anchored to the skull using two screws and dental cement.

Rats were allowed to recover in their cages for the following three days, and continued the feeding protocol (Fig 1).

After the last feeding on day 26, a microdialysis probe (MAB 4.15.2. Cut off: 6 kDa; 4 Cuprophane; 3 mm dialysis membrane) was placed into the right core region of the NAcc and perfused with artifical cerebrospinal fluid (aCSF: 145 mM NaCl; 1.25 mM CaCl_2_; 2.8 mM KCl; 1.2 mM MgCl_2_·6H_2_O; 1.25 mM NaH_2_PO_4_·H_2_O; pH 7.3; 2 μl/min). During the collection period, a balanced arm with a dual channel swivel was connected to the perfusion line and samples were gathered by an automated sample collector (CMA 140), which was maintained at 6°C. After collection, the perfusate was frozen at -80°C until further analysis. Cannulas, probes, and microdialysis equipment were provided by CMA (Solna, Sweden).

### Determination of the withdrawal onset time

**Fig 2:**
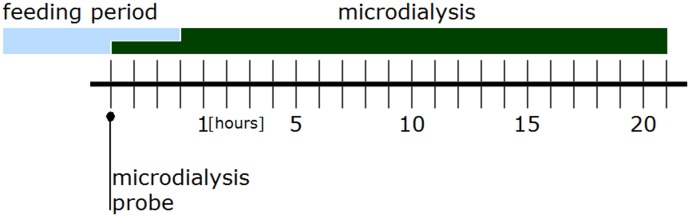
Experimental schedule for determining the withdrawal onset time.

In order to determine the time of onset of AWS, 10 rats randomly chosen from each of the alcohol and control groups were analyzed. The microdialysis probe was placed as described above, and microdialysate samples were collected at 60 minute intervals for 24 hours (Fig 2). The data collected during the first three hours were used to calculate baseline levels of the amino acids, and then nutrition was withheld. In line with recent studies [19,21], we defined the time at which we first observed a significant difference in glutamate levels between the alcohol and control groups as the “onset time of the withdrawal response”.

### Inhalational anesthesia during withdrawal

**Fig 3:**
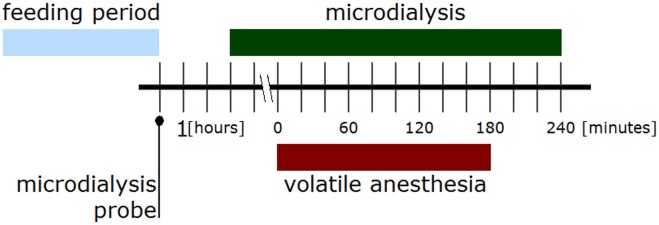
Experimental schedule for induction and microdialysis measurement of volatile anesthesia.

Thirty rats from each of the alcohol and control groups were used to study the effects of inhalational anesthesia on amino acid levels in the NAcc. Nutrition was withheld and microdialysis probes were inserted into position (Fig 3). After three hours of recovery microdialysate samples were collected. The first two hours of microdialysis measurements were used to generate the baseline. Experimental microdialysate samples were collected after this baseline period at 20 min intervals over a total period of 360 min. Anesthesia was induced in both groups five hours after food was withheld (matching the time point labeled “0” in Fig 3) in order to achieve a maximal withdrawal response, according to our findings from the experiment above. The thirty rats from each group were divided into three sub-groups which underwent inhalational anesthesia, receiving isoflurane, sevoflurane, or desflurane for 180 min, respectively, at a minimal alveolar concentration of 1. Body temperature was controlled using a heating pad and held at 37 ± 0.5°C, so as to prevent hypothermia.

Microdialysis was performed until one hour after emergence of anesthesia, in order to evaluate the elimination period of the anesthesia. After collection, perfusate was immediately frozen at -80°C until further analysis.

### Verification of probe position

Each animal was euthanized, then decapitated with a guillotine. To verify proper probe placement histologically, brains were first removed and preserved with 10% formalin in a 25% sucrose solution. For slicing, preserved brains were frozen on ice and then 100 μm thick coronal sections were cut with a vibratome and mounted on slides. Correct probe placement was verified in brains using a light microscope, according to the rat brain atlas of Paxinos and Watson [20].

### Amino acid analysis

The glutamate concentrations of microdialysate samples were analyzed, with samples collected during and after inhalational anesthesia being additionally analyzed for their aspartate, arginine, and GABA concentrations. Analysis was performed via reverse-phase high performance liquid chromatography (RP-HPLC; Bischoff Chromatography, Leonberg, Germany; and Merck, Darmstadt, Germany), using a fluorescence detector with an excitation wavelength of 330 nm and an emission cut-off filter at 450 nm. The reverse-phase ion-pairing column was prepacked with sulfonamidochrysoïdine (Prontosil) 120-3-C18 ace-EPS, 3 μm particulate surface 300 m^2^/g (Bischoff Chromatography, Leonberg, Germany). The mobile phase A contained 50 mM sodium acetate at pH 6.2 with 5% acetonitrile, and the mobile phase B contained 75% acetonitrile. The flow rate was 0.6 ml/min. The derivatization was performed automatically with a sampler maintained at 4°C (231 XL, Gilson, Middleton, Wisconsin). To identify the amino acids (glutamate, aspartate, arginine, and GABA), peaks were equalized to the standard peaks of these substrates. The detection limit of all amino acids was 0.05 pmol. Quantification was performed by integrating the area under the curve (AUC).

### Statistical analysis

The study in its entirety was planned and performed as an observational pilot study, meaning that the sample size was not statistically estimated beforehand.

Results are given as the mean with standard deviation [SD], or the median with 25% to 75% quartiles, in cases where the data deviated from a normal distribution. Due to the small sample sizes, only non-parametric tests were performed.

The absolute quantified amino acid concentrations from the AUC of the RP-HPLC analyses were expressed as percentages relative to the mean basal values, which were calculated as the mean of the three samples prior to the intervention. Microdialysis samples were collected at 60 minute intervals. The withdrawal onset time was defined as the earliest timepoint at which there was a significant difference between the alcohol and control groups, according to nonparametric Mann-Whitney U tests calculated at each timepoint.

In order to analyze inhalational anesthesia during withdrawal, microdialysis samples were collected at 20 min intervals for a total of 6 hours. The baseline was defined as the mean of the first six samples prior to anesthesia. Longitudinal data were analyzed using a two-way nonparametric repeated-measures MANOVA factorial design (1^st^, independent factor [treat]: alcohol and control groups; 2^nd^, dependent factor [time]: repetitions across time). This allowed us to analyze group differences between alcohol and control groups over time, including tests for differences between treatments [treat], systematic changes in time [time], and interactions between treatments and time [treat*time]. Post-hoc analyses were performed using Mann-Whitney U tests, in order to determine the timepoints at which significant differences occurred.

The data in the figures are expressed as percent of baseline, median with 25% to 75% percentiles, and time elapsed. All tests were performed as exploratory data analyses, and as such no adjustments for multiple testing have been made. The level of significance for these exploratory analyses was set at *p < 0.05, **p < 0.01 and ***p < 0.001. Numerical calculations were performed with IBM SPSS Statistics for Windows (Version 21.0; IBM Corp., Armonk, New York), and SAS 9.1 (SAS Institute, Inc., Cary, North Carolina).

## Results

### Exclusion rate

In order to determine the onset time of alcohol withdrawal, we randomly selected 20 rats (10 from the alcohol group and 10 controls). Due to technical difficulties and dislocation of microdialysis probes, only 14 rats could be included (8 rats in the control group and 6 in the alcohol group).

A total of 60 rats received inhalational anesthesia during withdrawal. Two animals died of unknown causes during recovery following implantation of the microdialysis cannula. Both rats belonged to the alcohol group, and had been previously assigned to the isoflurane and sevoflurane anesthesia sub-groups. Microdialysis data from two rats receiving desflurane, one in the alcohol group and the other in the control group, could not be analyzed due to technical difficulties, and therefore were excluded from further analyses.

In cases where a single microdialysis measurement was missing from an individual animal, we have disregarded data from that timepoint in the affected animal only.

### Animals and alcohol uptake

On the day of microdialysis, the average weight of the control rats was 296 g (SD = 15.6 g), compared to 277 g (SD = 17.6 g) for the AWR (p < 0.001). Before the stereotactic operation was performed, daily alcohol uptake was 2.21 g/day (SD = 0.46 g/day), which decreased to 1.70 g/day (SD = 0.5 g/day) between the operation and the beginning of microdialysis.

### Determination of the withdrawal onset time

**Fig 4:**
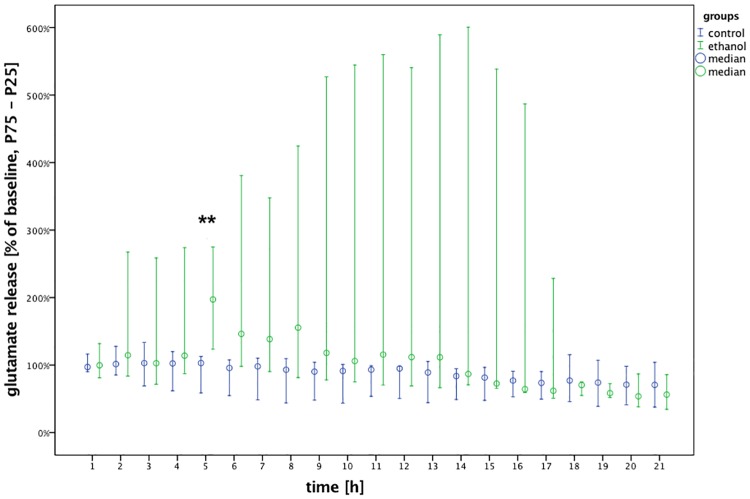
Elevated glutamate levels in the alcohol group compared to controls indicate that the alcohol withdrawal response occurred five hours after the last intake of alcohol. Glutamate release as median % of baseline with interquartile ranges (25–75%) plotted over time (control: n = 8; alcohol: n = 6). Group statistical analyses were performed using Mann-Whitney U tests (**p < 0.01).

Data collected from the alcohol and control group rats were compared at each timepoint (Fig 4). The baseline glutamate concentration was calculated as the mean of the values during the two hours before nutrition was withheld. The withdrawal response, defined as the time of the first significant difference in glutamate concentrations between the two groups, was detected five hours after food was withheld (p_5h_ = 0.002). Within the alcohol group, a few rats showed an earlier increase in glutamate release, resulting in a large interquartile range from the 2^nd^ hour onwards.

### Inhalational anesthesia during withdrawal

**Fig 5:**
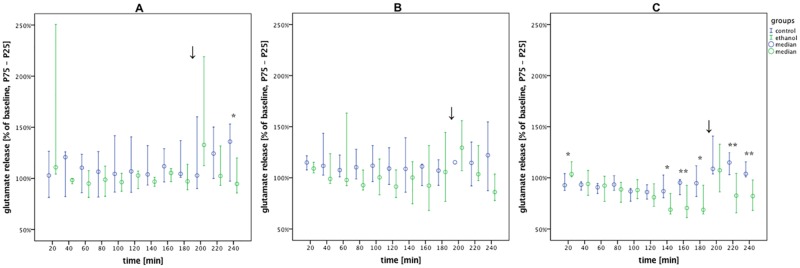
Glutamate release under desflurane was lower in AWR compared to controls for all timepoints from 140 mins onwards, except at 200 min. Glutamate release in the alcohol vs. control groups during withdrawal under volatile anesthesia (0–180 min), emergence (↓) and post-anesthesia recovery (200–240 min). Data are shown as median % of baseline with interquartile ranges (25–75%) and plotted over time. (A) isoflurane: alcohol vs. control, n = 9 vs. 10. (B) sevoflurane: alcohol vs. control, n = 9 vs. 10. (C) desflurane: alcohol vs. control, n = 9 vs. 9. Post-hoc univariate group comparisons were performed using Mann-Whitney U tests. (*p < 0.05 and **p < 0.01).

**Fig 6:**
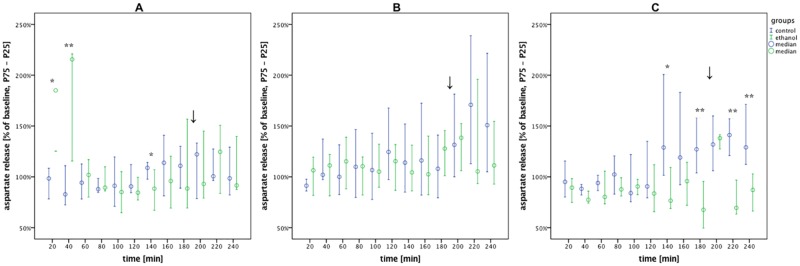
Aspartate levels under the different volatile anesthetics. Aspartate release in the alcohol vs. control groups during withdrawal under volatile anesthesia (0–180 min), emergence (↓) and post-anesthesia recovery (200–240 min). Data are shown as median % of baseline with interquartile ranges (25–75%) and plotted over time. (A) isoflurane: alcohol vs. control, n = 9 vs. 10. (B) sevoflurane: alcohol vs. control, n = 9 vs. 10. (C) desflurane: alcohol vs. control, n = 9 vs. 9. Post-hoc univariate group comparisons were performed using Mann-Whitney U tests (*p < 0.05 and **p < 0.01).

**Fig 7:**
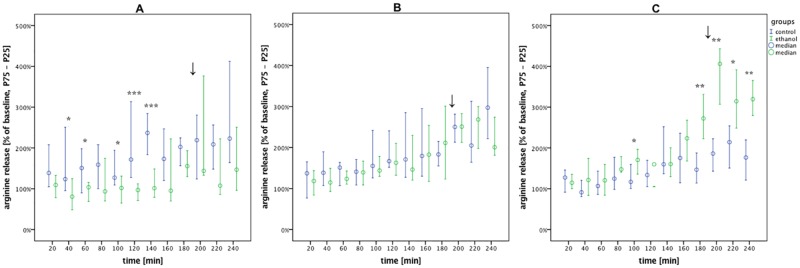
Arginine levels under the different volatile anesthetics. Arginine release in the alcohol vs. control groups during withdrawal under volatile anesthesia (0–180 min), emergence (↓) and post-anesthesia recovery (200–240 min). Data are shown as median % of baseline with interquartile ranges (25–75%) and plotted over time. (A) isoflurane: alcohol vs. control, n = 9 vs. 10. (B) sevoflurane: alcohol vs. control, n = 9 vs. 10. (C) desflurane: alcohol vs. control, n = 9 vs. 9. Post-hoc univariate group comparisons were performed using Mann-Whitney U tests. (*p < 0.05, **p < 0.01 and ***p < 0.001).

**Fig 8:**
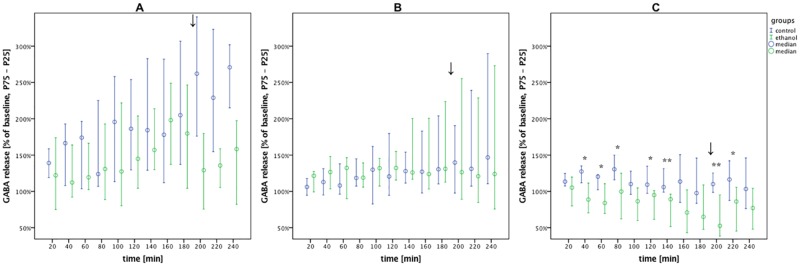
The GABA concentration was lower in AWR than controls under desflurane anesthesia. GABA release in the alcohol vs. control groups during withdrawal under volatile anesthesia (0–180 min), emergence (↓) and post-anesthesia recovery (200–240 min). Data are shown as median % of baseline with interquartile ranges (25–75%) and plotted over time (A) isoflurane: alcohol vs. control, n = 9 vs. 10. (B) sevoflurane: alcohol vs. control, n = 9 vs. 10. (C) desflurane: alcohol vs. control, n = 9 vs. 9. Post-hoc univariate group comparisons were performed using Mann-Whitney U tests (*p < 0.05 and **p < 0.01).

Each of the three volatile anesthetics (isoflurane, sevoflurane and desflurane) was administered to three sub-groups of rats from the alcohol and control groups. We did not perform any statistical comparisons of the relative effects of the different anesthetics. The baseline was calculated as the mean of the samples in the two hours prior to the onset of anesthesia. In the microdialysis plots in Figs 4–8, the starting value at 0 min is 100% for all groups (not plotted).

### Glutamate level

MANOVA factorial analyses revealed significant differences in glutamate concentration over time during isoflurane (p = 0.035), sevoflurane (p = 0.009), and desflurane (p < 0.001) anesthesia (Table 1). However at any of the individual timepoints during isoflurane and sevoflurane anesthesia, glutamate release in AWR and controls did not differ significantly (Fig 5). Under desflurane, glutamate concentration was elevated in AWR compared to controls at the first timepoint (20 min; p_20min_ = 0.049), but was lower for every timepoint after 140 min, except at 200 min (p_140min_ = 0.014, p_160min_ = 0.007, p_180min_ = 0.019, p_220min_ = p_240min_ = 0.007). A similar pattern was observed under isoflurane, as glutamate concentration was lower in AWR relative to controls at the final timepoint (p_240min_ = 0.023).

**Table 1:** P-values for differences in glutamate concentration from MANOVA analyses.

Hypotheses	Isoflurane: alcohol vs. control (p-values)	Sevoflurane: alcohol vs. control (p-values)	Desflurane alcohol vs. control (p-values)
[treat]	.42297	.30625	.08708
[time]	.03486	.00941	< 0.001
[treat*time]	.04791	.41992	< 0.001

Multivariate nonparametric analyses of longitudinal data were performed using a two-way factorial design (1^st^, independent factor [treat]: alcohol and control groups; 2^nd^, dependent factor [time]: repetitions in time; nonparametric MANOVA for repeated measures). P-values were calculated for comparison of treatment groups (alcohol vs. control) [treat], systematic changes in time [time], and interactions between treatment group and time [treat*time].

### Aspartate level

MANOVA factorial analyses showed that sevoflurane anesthesia caused systematic changes in aspartate concentration over time (p < 0.001) (Table 2). Under desflurane the MANOVA analysis revealed differences between alcohol and control groups (p = 0.004), with post-hoc tests showing significantly lower aspartate in AWR compared to controls at later timepoints (p_140min_ = 0.014, p_160min_ = p_220min_ = p_240min_ = 0.007, p_180min_ = 0.019). After induction of isoflurane anesthesia (Fig 6), aspartate release in AWR was increased at first compared to controls (p_20min_ = 0.015 and p_40min_ = 0.006), and subsequently dropped back to baseline levels. At one timepoint, the aspartate concentration in AWR fell below that of the control group (p_140min_ = 0.027).

**Table 2:** P-values for differences in aspartate concentration from MANOVA analyses.

p-values	Isoflurane: alcohol vs. control (p-values)	Sevoflurane: alcohol vs. control (p-values)	Desflurane: alcohol vs. control (p-values)
[treat]	.70391	.73800	.00401
[time]	.12853	.00025	.00071
[treat*time]	.01511	.43720	.00293

Multivariate nonparametric analyses of longitudinal data were performed using a two-way factorial design (1^st^, independent factor [treat]: alcohol and control groups; 2^nd^, dependent factor [time]: repetitions in time; nonparametric MANOVA for repeated measures). P-values show significance for group (alcohol vs. control) differences between treatment [treat], systematic changes in time [time], and interactions between treatment group and time [treat*time].

### Arginine level

Under all three of the volatile anesthetics, the release of arginine (Fig 7) showed significant systematic changes in time (p < 0.001) according to MANOVA analyses (Table 3). These analyses revealed significant differences between the alcohol and control groups under isoflurane (p = 0.002) and desflurane (p = 0.01).

**Table 3:** P-values for differences in arginine concentration from MANOVA analyses.

p-values	Isoflurane: alcohol vs. control (p-values)	Sevoflurane: alcohol vs. control (p-values)	Desflurane: alcohol vs. control (p-values)
[treat]	.00243	.56548	.00984
[time]	< 0.001	< 0.001	< 0.001
[treat*time]	.22394	.73548	.17936

Multivariate nonparametric analyses of longitudinal data were performed using a two-way factorial design (1^st^, independent factor [treat]: alcohol and control groups; 2^nd^, dependent factor [time]: repetitions in time; nonparametric MANOVA for repeated measures). P-values show significance for group (alcohol vs. control) differences between treatment [treat], systematic changes in time [time], and interactions between treatment group and time [treat*time].

Post-hoc analyses showed that during isoflurane anesthesia, arginine release in AWR was lower than the control group at the majority of the timepoints measured (p_40min_ = 0.035, p_60min_ = 0.036, p_100min_ = 0.027, p_120min_ < 0.001, p_140min_ < 0.001). In contrast, desflurane led to an elevated release of arginine in AWR compared to controls at several timepoints (p_100min_ = 0.014, p_180min_ = 0.003, p_200min_ = 0.008, p_220min_ = 0.012, p_240min_ = 0.009).

### GABA level

MANOVA analyses showed that isoflurane caused systematic changes in GABA concentrations over time (p = 0.002) (Table 4). The MANOVA revealed significant differences between the alcohol and control groups under desflurane (p = 0.023), with post-hoc analyses on individual timepoints (Fig 8) showing that GABA release was lower in AWR than controls both during and after desflurane anesthesia (p_40min_ = p_60min_ = 0.019, p_80min_ = 0.015, p_120min_ = 0.026, p_140min_ = p_200min_ = 0.009, p_220 min_ = 0.041).

**Table 4:** P-values for differences in GABA concentration from MANOVA analyses.

p-values	Isoflurane: alcohol vs. control (p-values)	Sevoflurane: alcohol vs. control (p-values)	Desflurane: alcohol vs. control (p-values)
[treat]	.11200	.79603	.02314
[time]	.00222	.22409	.06374
[treat*time]	.05414	.60808	.77384

Multivariate nonparametric analyses of longitudinal data were performed using a two-way factorial design (1^st^, independent factor [treat]: alcohol and control groups; 2^nd^, dependent factor [time]: repetitions in time; nonparametric MANOVA for repeated measures). P-values show significance for group (alcohol vs. control) differences between treatment [treat], systematic changes in time [time], and interactions between treatment group and time [treat*time].

## Discussion

Our findings showed that glutamate concentrations increase after alcohol withdrawal in rats with chronic alcohol uptake (Fig 4), in agreement with many published studies [19,21–23].

The onset time of the alcohol withdrawal response was defined as the earliest timepoint at which we measured a significant difference in glutamate levels between the alcohol and control groups in the core region of the NAcc. This method has been used previously in similar experimental designs [19]. Although interindividual variability was broad, this response was found to take place around five hours after the last oral alcohol uptake, which is consistent with findings from other research groups [19,24].

To synchronize with this withdrawal response, in the second trial volatile anesthesia was induced five hours after the last oral alcohol uptake, when a significant release of glutamate was expected. During anesthesia, there were no significant differences in glutamate concentration between the alcohol and control groups under isoflurane or sevoflurane, although there was a large variability in the first timepoint measured in AWR under isoflurane. A modest increase in glutamate release in AWR was seen immediately after induction with desflurane. However at later timepoints, glutamate release in AWR decreased below that in controls.

Analogous to glutamate release, aspartate release of AWR in the desflurane sub-group showed a tendency to be lower over the entire timecourse of microdialysis, with significant differences apparent from 140 min after induction. Under sevoflurane anesthesia, there were no apparent group differences in aspartate release. Induction of isoflurane caused an increase in aspartate release in AWR relative to controls for the first 40 minutes of anesthesia.

Aspartate is an excitatory neurotransmitter of the NMDA receptor [25], but the excitotoxicity seems to be less severe in comparison to glutamate [26]. Both glutamate and aspartate, the excitatory amino acids we measured in this study, are absorbed out of the synaptic gap mostly due to excitatory amino acid transporters (EAAT). These transporters are the most important regulators of excitatory neurotransmission [27], but their function is reduced under chronic alcohol exposure [28], leading to impaired glutamate uptake during withdrawal.

The different EAAT are thought to be partly responsible for the varying effects of each of the volatile anesthetics, but the precise molecular pathways underlying these effects remain unclear. Volatile anesthetics reduce exocytosis [29,30], and increase excitatory transmitter uptake [31,32]. An increased activity of EAAT3 has been shown under isoflurane, sevoflurane, and desflurane anesthesia [30,33–36], and is mediated by activated proteinkinase C (PKC) [35]. PKC plays a central regulatory role via potentiation of NMDA receptor trafficking [37].

It is remarkable that neuronal structures adapted to alcohol seem to respond more sensitively to volatile anesthesia, and are more efficient in reducing excitatory neurotransmitters than those that are not adapted.

Under each volatile anesthetic, our data showed an increase in arginine release over the entire microdialysis timecourse, both in AWR and alcohol-naïve rats. When compared to the control group, the release of arginine in the AWR group was lower under isoflurane, equal under sevoflurane, and higher under desflurane. GABA release under isoflurane increased over the timecourse, according to the known mode of action. However under sevoflurane, we did not measure any increase in GABA release. Under desflurane, GABA release was lower in AWR than controls at the majority of the timepoints measured. These results might be due to limitations in the microdialysis approach, since the potentiation of GABA-induced currents [38] cannot be measured by this method.

### Limitations to the Study

Since this study was performed as a pilot study, we did not statistically estimate the sample size beforehand. A possible consequence of this is that the data sample collected was not sufficiently large to show significant differences for particular comparisons.

The spread of data in the alcohol group may reflect variability due to differences in individual metabolism and alcohol uptake behavior from the unforced uptake protocol, which we used based on the recommendations of the institutional animal care board. Comparable studies with strict, regular feeding procedures, such as intragastric feeding or intraperitoneal application, showed lower variability but a similar onset time for withdrawal.

In this study, every anesthetic treatment group was matched with its own control group. This method is not suitable for comparing the treatment groups, and so direct comparisons of the three anesthetics to each other cannot be made.

Although sevoflurane and desflurane are routinely used in clinical practice, only a few experimental studies have been published which investigate the mode of action and effects of both anesthetics. Isoflurane is predominantly used in such experiments, although it is rarely used in clinical practice in industrialized nations. This fact makes a reliable translation of our results to human clinical practice difficult.

Volatile anesthetics have an alcohol-like effect on the NMDA receptor through their action as noncompetitive antagonists [39], and isoflurane even leads to a compensatory increase in expression of the NMDA subunit NR2B [40]. To evaluate the total inhibitory effect of the anesthetics, it is necessary to consider not only the concentration of transmitters, but also their induced currents and postsynaptic effects. The microdialysis approach alone is not sufficient to measure such effects.

## Conclusion

In this study, we showed an increased glutamate release in AWR during alcohol withdrawal compared to controls. Desflurane induction led to a moderate glutamate increase in AWR, whereas aspartate and GABA levels gradually decreased over the timecourse, following the increase in glutamate release. Under isoflurane, aspartate release was elevated in AWR compared to controls after induction, whilst the controls showed higher arginine concentrations. There were no group differences under sevoflurane anesthesia. All three anesthetics intensified the release of arginine over the timecourse.

Due to the complexity and lack of clarity regarding the mechanisms of action of the anesthetics, presenting reliable explanations for the effects we observed would be challenging. Since EAAT are the most important known regulators of excitatory neurotransmission, their differing affinities with each of the volatile anesthetics used in this study may explain their variable effects, and could be a good starting point for further research.

## Supporting Information

S1 Table Baseline statistics. (XLSX)

## References

[1] Mehta AJ. Alcoholism and critical illness: A review. World J Crit Care Med. 2016;5: 27–35. 10.5492/wjccm.v5.i1.27 26855891 PMC4733453

[2] Hasin DS, Stinson FS, Ogburn E, Grant BF. Prevalence, correlates, disability, and comorbidity of DSM-IV alcohol abuse and dependence in the United States: results from the National Epidemiologic Survey on Alcohol and Related Conditions. Arch Gen Psychiatry. 2007;64: 830–842. 10.1001/archpsyc.64.7.830 17606817

[3] Spies CD, Neuner B, Neumann T, Blum S, Müller C, Rommelspacher H, et al Intercurrent complications in chronic alcoholic men admitted to the intensive care unit following trauma. Intensive Care Med. 1996;22: 286–293. 8708164 10.1007/BF01700448

[4] Trevisan LA, Boutros N, Petrakis IL, Krystal JH. Complications of alcohol withdrawal: pathophysiological insights. Alcohol Health Res World. 1998;22: 61–66. 15706735 PMC6761825

[5] Eyer F, Schuster T, Felgenhauer N, Pfab R, Strubel T, Saugel B, et al Risk assessment of moderate to severe alcohol withdrawal—predictors for seizures and delirium tremens in the course of withdrawal. Alcohol Alcohol. 2011;46: 427–433. 10.1093/alcalc/agr053 21593124

[6] Quintero GC. Role of nucleus accumbens glutamatergic plasticity in drug addiction. Neuropsychiatr Dis Treat. 2013;9: 1499–1512. 10.2147/NDT.S45963 24109187 PMC3792955

[7] Fliegel S, Brand I, Spanagel R, Noori HR. Ethanol-induced alterations of amino acids measured by in vivo microdialysis in rats: a meta-analysis. In Silico Pharmacol. 2013;1: 7 10.1186/2193-9616-1-7 25505652 PMC4230485

[8] Chen G, Cuzon Carlson VC, Wang J, Beck A, Heinz A, Ron D, et al Striatal involvement in human alcoholism and alcohol consumption, and withdrawal in animal models. Alcohol Clin Exp Res. 2011;35: 1739–1748. 10.1111/j.1530-0277.2011.01520.x 21615425 PMC3276303

[9] Nath V, Reneau JC, Dertien JS, Agrawal RG, Guerra I, Bhakta Y, et al An in vitro model for studying the effects of continuous ethanol exposure on N-methyl-D-aspartate receptor function. Alcohol. 2012;46: 3–16. 21925827 10.1016/j.alcohol.2011.08.003 PMC3258339

[10] Hendricson AW, Maldve RE, Salinas AG, Theile JW, Zhang TA, Diaz LM, et al Aberrant synaptic activation of N-methyl-D-aspartate receptors underlies ethanol withdrawal hyperexcitability. J Pharmacol Exp Ther. 2007;321: 60–72. 10.1124/jpet.106.111419 17229881

[11] Moore RD, Bone LR, Geller G, Mamon JA, Stokes EJ, Levine DM. Prevalence, detection, and treatment of alcoholism in hospitalized patients. JAMA. 1989;261: 403–407. 2909780

[12] Awissi D-K, Lebrun G, Coursin DB, Riker RR, Skrobik Y. Alcohol withdrawal and delirium tremens in the critically ill: a systematic review and commentary. Intensive Care Med. 2013;39: 16–30. 10.1007/s00134-012-2758-y 23184039

[13] Simms JA, Steensland P, Medina B, Abernathy KE, Chandler LJ, Wise R, et al Intermittent access to 20% ethanol induces high ethanol consumption in Long-Evans and Wistar rats. Alcohol Clin Exp Res. 2008;32: 1816–1823. 10.1111/j.1530-0277.2008.00753.x 18671810 PMC3151464

[14] Cippitelli A, Damadzic R, Singley E, Thorsell A, Ciccocioppo R, Eskay RL, et al Pharmacological blockade of corticotropin-releasing hormone receptor 1 (CRH1R) reduces voluntary consumption of high alcohol concentrations in non-dependent Wistar rats. Pharmacol Biochem Behav. 2012;100: 522–529. 10.1016/j.pbb.2011.10.016 22036774 PMC3242882

[15] Lieber CS, DeCarli LM. Liquid diet technique of ethanol administration: 1989 update. Alcohol Alcohol. 1989;24: 197–211. 2667528

[16] Mucha RF, Pinel JP, Oot PH. Simple method for producing an alcohol withdrawal syndrome in rats. Pharmacol Biochem Behav. 1975;3: 765–769. 1239771 10.1016/0091-3057(75)90104-5

[17] Sancho-Tello M, Muriach M, Barcia J, Bosch-Morell F, Genovés JM, Johnsen-Soriano S, et al Chronic alcohol feeding induces biochemical, histological, and functional alterations in rat retina. Alcohol Alcohol. 2008;43: 254–260. 10.1093/alcalc/agn006 18304993

[18] McBride WJ, Li TK. Animal models of alcoholism: neurobiology of high alcohol-drinking behavior in rodents. Crit Rev Neurobiol. 1998;12: 339–369. 10348615 10.1615/critrevneurobiol.v12.i4.40

[19] Saellstroem Baum S, Huebner A, Krimphove M, Morgenstern R, Badawy AA-B, Spies CD. Nicotine stimulation on extracellular glutamate levels in the nucleus accumbens of ethanol-withdrawn rats in vivo. Alcohol Clin Exp Res. 2006;30: 1414–1421. 10.1111/j.1530-0277.2006.00169.x 16899045

[20] Paxinos G, Watson C. The Rat Brain in Stereotaxic Coordinates—The New Coronal Set. Academic Press; 2004.

[21] Christian DT, Alexander NJ, Diaz MR, McCool BA. Thalamic glutamatergic afferents into the rat basolateral amygdala exhibit increased presynaptic glutamate function following withdrawal from chronic intermittent ethanol. Neuropharmacology. 2013;65: 134–142. 10.1016/j.neuropharm.2012.09.004 22982568 PMC3521082

[22] Dahchour A, De Witte P. Taurine blocks the glutamate increase in the nucleus accumbens microdialysate of ethanol-dependent rats. Pharmacol Biochem Behav. 2000;65: 345–350. 10672989 10.1016/s0091-3057(99)00197-5

[23] Dahchour A, De Witte P. Excitatory and inhibitory amino acid changes during repeated episodes of ethanol withdrawal: an in vivo microdialysis study. Eur J Pharmacol. 2003;459: 171–178. 12524143 10.1016/s0014-2999(02)02851-0

[24] Rossetti ZL, Carboni S, Fadda F. Glutamate-induced increase of extracellular glutamate through N-methyl-D-aspartate receptors in ethanol withdrawal. Neuroscience. 1999;93: 1135–1140. 10473277 10.1016/s0306-4522(99)00250-x

[25] Nadler JV. Aspartate release and signalling in the hippocampus. Neurochem Res. 2011;36: 668–676. 10.1007/s11064-010-0291-3 20953700

[26] Chen PE, Geballe MT, Stansfeld PJ, Johnston AR, Yuan H, Jacob AL, et al Structural features of the glutamate binding site in recombinant NR1/NR2A N-methyl-D-aspartate receptors determined by site-directed mutagenesis and molecular modeling. Mol Pharmacol. 2005;67: 1470–1484. 10.1124/mol.104.008185 15703381

[27] Mathews GC, Diamond JS. Neuronal glutamate uptake Contributes to GABA synthesis and inhibitory synaptic strength. J Neurosci. 2003;23: 2040–2048. 12657662 10.1523/JNEUROSCI.23-06-02040.2003 PMC6742021

[28] Kim J-H, Do S-H, Kim Y-L, Zuo Z. Effects of chronic exposure to ethanol on glutamate transporter EAAT3 expressed in Xenopus oocytes: evidence for protein kinase C involvement. Alcohol Clin Exp Res. 2005;29: 2046–2052. 16340463 10.1097/01.alc.0000187594.92476.07

[29] Ouyang W, Hemmings HC. Depression by isoflurane of the action potential and underlying voltage-gated ion currents in isolated rat neurohypophysial nerve terminals. J Pharmacol Exp Ther. 2005;312: 801–808. 10.1124/jpet.104.074609 15375177

[30] Moe MC, Berg-Johnsen J, Larsen GA, Røste GK, Vinje ML. Sevoflurane reduces synaptic glutamate release in human synaptosomes. J Neurosurg Anesthesiol. 2002;14: 180–186. 12172289 10.1097/00008506-200207000-00002

[31] Larsen M, Langmoen IA. The effect of volatile anaesthetics on synaptic release and uptake of glutamate. Toxicol Lett. 1998;100–101: 59–64. 10049181 10.1016/s0378-4274(98)00165-9

[32] Larsen M, Hegstad E, Berg-Johnsen J, Langmoen IA. Isoflurane increases the uptake of glutamate in synaptosomes from rat cerebral cortex. Br J Anaesth. 1997;78: 55–59. 9059205 10.1093/bja/78.1.55

[33] Lee S-A, Choi J-G, Zuo Z. Volatile anesthetics attenuate oxidative stress-reduced activity of glutamate transporter type 3. Anesth Analg. 2009;109: 1506–1510. 19843789 10.1213/ANE.0b013e3181b6709a PMC2773695

[34] Huang Y, Feng X, Sando JJ, Zuo Z. Critical role of serine 465 in isoflurane-induced increase of cell-surface redistribution and activity of glutamate transporter type 3. J Biol Chem. 2006;281: 38133–38138. 10.1074/jbc.M603885200 17062570

[35] Do S-H, Kamatchi GL, Washington JM, Zuo Z. Effects of volatile anesthetics on glutamate transporter, excitatory amino acid transporter type 3: the role of protein kinase C. Anesthesiology. 2002;96: 1492–1497. 12170065 10.1097/00000542-200206000-00032

[36] Lee SN, Li L, Zuo Z. Glutamate transporter type 3 knockout mice have a decreased isoflurane requirement to induce loss of righting reflex. Neuroscience. 2010;171: 788–793. 10.1016/j.neuroscience.2010.09.044 20875840 PMC3401886

[37] Yan J-Z, Xu Z, Ren S-Q, Hu B, Yao W, Wang S-H, et al Protein kinase C promotes N-methyl-D-aspartate (NMDA) receptor trafficking by indirectly triggering calcium/calmodulin-dependent protein kinase II (CaMKII) autophosphorylation. J Biol Chem. 2011;286: 25187–25200. 10.1074/jbc.M110.192708 21606495 PMC3137090

[38] Hapfelmeier G, Schneck H, Kochs E. Sevoflurane potentiates and blocks GABA-induced currents through recombinant alpha1beta2gamma2 GABAA receptors: implications for an enhanced GABAergic transmission. Eur J Anaesthesiol. 2001;18: 377–383. 11412290 10.1046/j.0265-0215.2001.00848.x

[39] Hollmann MW, Liu HT, Hoenemann CW, Liu WH, Durieux ME. Modulation of NMDA receptor function by ketamine and magnesium. Part II: interactions with volatile anesthetics. Anesth Analg. 2001;92: 1182–1191. 11323344 10.1097/00000539-200105000-00020

[40] Mawhinney LJ, de Rivero Vaccari JP, Alonso OF, Jimenez CA, Furones C, Moreno WJ, et al Isoflurane/nitrous oxide anesthesia induces increases in NMDA receptor subunit NR2B protein expression in the aged rat brain. Brain Res. 2012;1431: 23–34. 10.1016/j.brainres.2011.11.004 22137658 PMC3246550

